# Analyzing Multivariate Dynamics Using Cross-Recurrence Quantification Analysis (CRQA), Diagonal-Cross-Recurrence Profiles (DCRP), and Multidimensional Recurrence Quantification Analysis (MdRQA) – A Tutorial in R

**DOI:** 10.3389/fpsyg.2018.02232

**Published:** 2018-12-04

**Authors:** Sebastian Wallot, Giuseppe Leonardi

**Affiliations:** ^1^Max Planck Institute for Empirical Aesthetics, Frankfurt am Main, Germany; ^2^Faculty of Psychology, University of Economics and Human Sciences, Warsaw, Poland

**Keywords:** RQA, cross-recurrence quantification analysis, diagonal cross-recurrence profile, multidimensional recurrence quantification analysis, R, tutorial

## Abstract

This paper provides a practical, hands-on introduction to cross-recurrence quantification analysis (CRQA), diagonal cross-recurrence profiles (DCRP), and multidimensional recurrence quantification analysis (MdRQA) in R. These methods have enjoyed increasing popularity in the cognitive and social sciences since a recognition that many behavioral and neurophysiological processes are intrinsically time dependent and reliant on environmental and social context has emerged. Recurrence-based methods are particularly suited for time-series that are non-stationary or have complicated dynamics, such as longer recordings of continuous physiological or movement data, but are also useful in the case of time-series of symbolic data, as in the case of text/verbal transcriptions or categorically coded behaviors. In the past, they have been used to assess changes in the dynamics of, or coupling between physiological and behavioral measures, for example in joint action research to determine the co-evolution of the behavior between individuals in dyads or groups, or for assessing the strength of coupling/correlation between two or more time-series. In this paper, we provide readers with a conceptual introduction, followed by a step-by-step explanation on how the analyses are performed in R with a summary of the current best practices of their application.

## Introduction

The assessment of behavioral and physiological dynamics, as well as the time-dependent coupling of physiological and behavioral activities were always of interest to the behavioral and social sciences. However, the proper assessment of dynamics and coupling has gained increased prominence in recent years in at least two areas of inquiry: One is the multidimensional assessment of physiological processes, i.e., physiological markers of emotional processes, after it had become generally accepted that no single feature (e.g., level of activity) of individual physiological measures (e.g., heart rate) are fully transparent to specific subjective states of arousal and emotion (see Kreibig, 2010, for a review). The other is research on joint action, where the goal is to understand online dynamic behavior of dyads or groups, and how their joint behavior influences subjective perceptions of the group members as well as their performance (see Marsh et al., 2009; Knoblich et al., 2011, for reviews).

What is common across these two domains of research is that they usually deal with multiple time-series (i.e., multiple physiological indicators or behavioral measures from multiple group members) that are often non-stationary, or possess other interesting dynamics. In the area of research on joint action, one prominent class of analysis methods that has been used increasingly in the last 10–15 years consists of several methods originating from the analysis of Recurrence Plots (RP; e.g., Shockley et al., 2003, 2007; Richardson and Dale, 2005; Dale et al., 2011b; Abney et al., 2014; Fusaroli and Tylén, 2016; Mønster et al., 2016; Wallot et al., 2016a,b; Vink et al., 2017).

This class of recurrence-based methods, while it has its roots in dynamical systems analysis and physics, is quickly emerging as a robust general-purpose technique to quantify order and organization (Webber et al., 2009), usually (but not only) in time-dependent signals and behaviors, and it has already found its way into several scientific fields. We can characterize it as a class of multivariate and generalized correlational analyses, that are suited for joint action data because they make very few assumptions and are particularly robust in case of non-linearities, non-stationary dynamics, and time-series with extreme outliers (Webber and Zbilut, 2005; Marwan et al., 2007; Fusaroli et al., 2014). For those reasons, they also have proven very robust to analyze data from joint action phenomena in semi-experimental and naturalistic settings, such as doctor-patient conversations (Angus et al., 2012), natural conversation between parents and children (Dale and Spivey, 2006), interaction between mothers and infants (Reddy et al., 2013; Nomikou et al., 2016; Abney et al., 2017), or shared emotional states during social rituals (Konvalinka et al., 2011). Apart from gaging the ordered, coordinated features of behaviors in time *per se*, the methods have the potential to shed lights into the transitions from an ordered state to another (Trulla et al., 1996), and hence to allow the mapping of singularities and characterizing instabilities in coordinated states and phase transitions (see e.g., Zbilut et al., 2002; Stephen et al., 2009a,b; Lichtwarck-Aschoff et al., 2012).

While these analyses have not received equal prominence in biopsychological research, many applications exists in the area of physiology (e.g., Webber and Zbilut, 1994; Webber et al., 1995; Dabiré et al., 1998; Giuliani et al., 1998; Zbilut et al., 2002; Marwan et al., 2005; Javorka et al., 2008; Schinkel et al., 2009; Acharya et al., 2011; Andrade et al., 2012; Fisher et al., 2015) and movement research (e.g., Kiefer et al., 2011; Ramenzoni et al., 2011).

Particularly, the multivariate methods we will introduce in the following sections allow to analyze non-stationary time-series and to assess how strongly two time-series are correlated, whether they exhibit leader-follower relationships (i.e., one measure following the time-course of the other by a certain lag), or how multiple (*n* > 2) variables evolve together over time. Hence, these methods can be used to assess levels and types of synchrony, coupling and entrainment of signals, and whether these change as a function of experimental manipulations – for example, in many joint action studies relevant task variables are manipulated (such as cooperative style, induced emotion, or social role), and their effects on degrees of behavioral or physiological synchronization are examined. In turn, synchronization properties are usuall used as mediating variables to predict subjective and objective task outcomes, such a quality and quantity of performance, joy or satisfaction. Hence, these methods can be used to for exploratory purposes, examining whether synchronization – or other kinds of coordination happens, or to test specific hypothesis about behavioral of physiological coordination under different conditions.

One reason why recurrence-based analyses are perhaps not equally prominent across all fields of research that deal with multivariate time-series is the lack of courses taught at Universities to students of the respective disciplines, but also the lack of implementation of the techniques in standard statistical software packages (such as STATA, JMP, SPSS, etc.). Several applications exist in C, MatLab, Python, R or other languages (an overview over different software packages can be found at *www.recurrence-plot.tk*, hosted by Norbert Marwan), but not all potentially interested users are familiar with those programming languages. However, in R a few packages implementing recurrence-based analyses are available for univariate and multivariate data analysis.

The aim of the current paper is to give a hands-on introduction to multivariate recurrence-based methods. We will specifically focus on the analysis of bivariate dynamics/ coordinated time-series (cross-recurrence quantification analysis; CRQA – Zbilut et al., 1998; Marwan and Kurths, 2002), on the analysis of leader-follower relations in bivariate time-series (diagonal cross-recurrence profile; DCRP – Richardson and Dale, 2005), and on the analysis of multivariate dynamics (multidimensional recurrence quantification analysis; MdRQA – Wallot et al., 2016b). We chose to present this tutorial in R because of the increasing popularity and availability of this software due to its free-ware nature, and the basic familiarity of many researchers with this environment.

In the following, we will first provide a brief overview over the central concepts of recurrence-based analyses and (auto) RQA, i.e., the analysis of a single time-series, together with the central steps in conducting the analyses. RQA is not at the core of this paper, as we are specifically interested in multivariate applications. However, presenting RQA briefly in the beginning helps to explain the concept of recurrence and the steps in conducting these analyses in general. Thereafter, we will provide a detailed hands-on introduction for conducting each of the three analysis techniques, presenting the necessary R-commands step-by-step. While CRQA and DCRP can be conducted using the *crqa()*- and *drpdfromts()*-function form the ‘crqa’ package (Coco and Dale, 2014), we provide readers with a new R-function for MdRQA in the Supplementary Materials to this paper, which has only been available in MatLab so far (Wallot et al., 2016b). At the end of each section, we discuss common issues of the analyses and the current best practice. Finally, we close the article with remarks and suggestions on how to use these analyses in sample comparisons.

## Recurrence-Based Analyses

As the name implies, the core-concept of recurrence-based analyses is *recurrence* – repetition of elements or patterns in a sequence. The core tool of these analyses is the recurrence plot or recurrence matrix, which is a means of displaying and charting repetitions in a sequence. As we will see further below, the analyses are usually not performed on the original 1-dimensional sequence or time-series, but on its phase-space portrait, which we will describe in more detail below. However, all of the analyses can be performed on the raw data. We will return to this issue later at the end of the section on parameter estimation.

**FIGURE 1:**
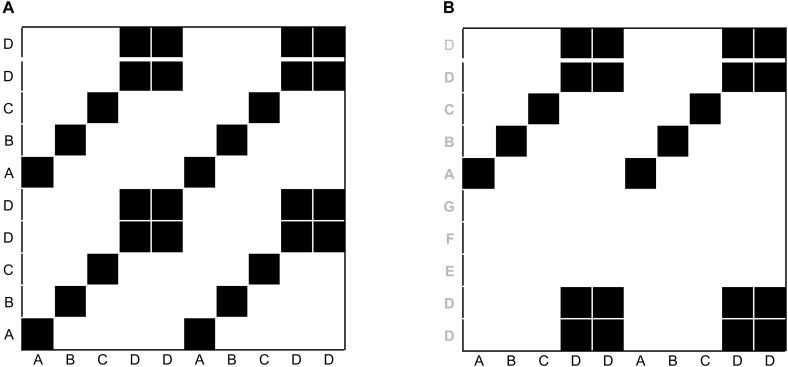
Illustration of recurrence of letters in the sequence “ABCDDABCDD” **(A)**, and cross-recurrence of letters in the sequence “ABCDDABCDD” with “DDEFGABCDD” **(B)**. The black squares in the matrices indicate the recurrence of a letter, and white spaces indicate the absence of recurrence. The distribution of recurrent points on the recurrence plot can be quantified to yield statistics of the repetitive patterns in a sequence.

Before we introduce particular variants of recurrence-based analyses, we want to briefly show how the recurrence plot captures repeating patterns in a sequence. Even though we are not interested in auto-recurrence analysis of a single time-series in this tutorial, we will briefly start with such an example in order to illustrate the concept of the recurrence plot. This can be easily illustrated using a simple, short 1-dimensional nominal sequence, “ABCDDABCDD.” The sequence is arbitrary (it could represent a series of coded behaviors of a participant over time), but is clearly not random, containing repetitive sub-sequences. A recurrence plot can be used to visualize these repeating characteristics by comparing all the elements of such a sequence with themselves, when aligned in the two dimensions of the plot (see Figure 1A).

The recurrence plot in Figure 1A needs not be limited to recurrences within the same single sequence. In other words, similar to the arbitrary letter-sequence presented above, we can extend the concept of the auto-recurrence plot of a single sequence and create a cross-recurrence plot, which examines cross-recurrences between two sequences, as in Figure 1B. Note that while the recurrence plot in Figure 1A possesses a diagonal of recurrent points (which simply means that every element in the sequence is recurrent with itself at lag 0), the cross-recurrence plot in Figure 1B does not necessarily possess such a diagonal. Moreover, while the recurrence plot is fully symmetrical about its main diagonal, this is not the case for the cross-recurrence plot. This is, because the two sequences in Figure 1B are not identical, but rather share only parts of their elements and sequential characteristics.

The cross-recurrence plot is not just a useful tool to *visualize* or *display* the sequential similarity of two sequences, but can be used to *quantify* their similarity. For example, the tally of all recurrent points on the plot tells us something about the repetitiveness of the individual elements across the two sequences, and we refer to this quantity as percent recurrence (*%REC*). Counting all recurrent points that have other, diagonally adjacent recurrent points and dividing them by *%REC* tells us something about the degree to which elements that occur in one sequence as larger, connected patterns also occur in the other sequence in the same order. This quantity is called percent determinism (*%DET*). Counting the average length of all diagonal lines of cross-recurrence points tells us something about the average size of the shared patterns (Average Diagonal Line, *ADL*). However, there are many more ways to quantify the (cross-)recurrence plot, and Table 1 charts the most common measures and their definition. All of them provide us with information of how the two sequences are similar or correlated.

**Table 1:** The most common cross-recurrence measures.

Variable name	Definition	Quantifies…
Percent Recurrence (%*REC*)	Sum of recurrent points in RP / Size of RP	…repetition of elements across the two sequences.
Percent Determinism (%*DET*)	Sum of diagonally adjacent recurrent points / Sum of recurrent points in RP	…how many of the individual repetitions co-occur in connected trajectories.
Average Diagonal Line Length (*ADL*)	Average diagonal lines in RP	…how long the average cross-repeating trajectory is.
Maximum Diagonal Line Length (*MDL*)	Length of longest diagonal line in RP (excluding the main diagonal)	…how long the longest cross-repeating trajectory is.


*Further RQA measures exist, and others are being developed – for the description of additional measure, see for example Marwan et al. (2007).*

As the application to the toy nominal data shows, recurrence-based analyses are very versatile. They can be used to compare sequences or time-series of nominal, ordinal, or interval-scale data. They make no assumptions about the distribution of data points, and are robust in the face of outliers and non-stationarity. However, they require multiple data points. Depending on the data at hand, the analyses can be used with as few as 10–30 data points, the upper limit being only set by the computational power accessible. However, we will return to data requirements in more detail at the end of the paper.

In the following section, we will apply the basic concept of recurrence and the recurrence plot to coupled time-series, especially multivariate cases where the correlation of the common dynamics of two (or more) time-series are of interest. Before that though, we will introduce the basic steps of parameter estimation that are necessary to conduct multivariate recurrence-based analyses, and reconstruct the multidimensional phase-space of a 1-dimensional time-series, on which the recurrence plot is computed.

## R Packages Needed

The following R packages – as well as their dependencies – need to be installed and loaded to run the tutoril: ‘crqa,’ ‘entropy,’ ‘nonlinearTseries,’ ‘plot3D,’ ‘SDMTools,’ and ‘tseriesChaos.’ The *mdrqa()*-function for the computation of MdRQA is not included in any *R*-package yet, but can be found in the Supplementary Materials to this paper.

## Generating Example Data

Here, our primary aim is to show how different recurrence-based analyses can be used to analyze multivariate dynamics, meaning the evolution of a single system that is distributed across multiple interdependent observables, or the evolution of different systems, but whose behaviors are coupled. To that end, we want to use the Lorenz-system (Lorenz, 1963), which is a system of three coupled differential equations (eq. 1):

$$\begin{array}{c} \overset{˙}{x}=\sigma \left(y-x\right)\\ \overset{˙}{y}=x(\rho -z)-y\\ \overset{˙}{z}=xy-\beta z \end{array}$$

The variables *x*, *y* and *z* are the three dimensions of the system that originally modeled hydrodynamic features, where the three dimensions represent the rotation rate in the fluid and two temperature measures (Lorenz, 1963). The parameters σ, ρ, and β are coupling parameters. Here, we are interested in interpreting the system as an analogy to human behavior. For example, *x*, *y*, and *z* could be measures of skin conductance (or any other quantity) of three members of a group that work together, and each member’s level of skin conductance at a certain time is not only determined by their idiosyncratic history, but also by interactions with the other members (i.e., future values of *x* do not only depend on past values of *x*, but also on past values of *y*), perhaps capturing changes of joint arousal within groups (e.g., Mønster et al., 2016).

**FIGURE 2:**
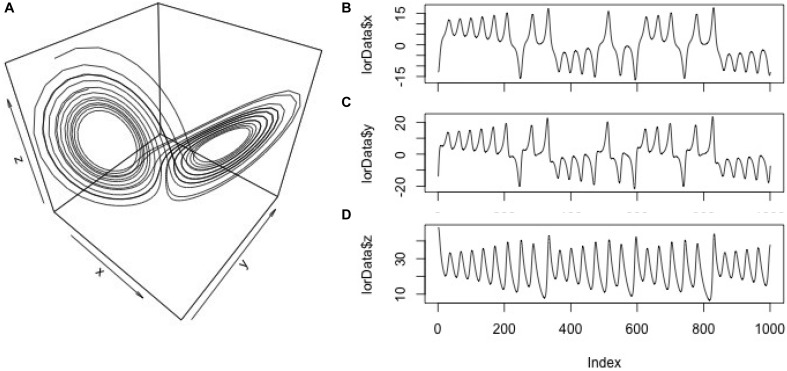
Display of the data. 3D-plot (a.k.a. phase-space) of the Lorenz system **(A)**; component time-series for the *x*, *y*, and *z*-axis of the Lorenz system **(B–D)**.

We resort to this artificial system for illustrational purposes, because it is a classic example for the behavior of a multidimensional system whose component-behaviors are interdependent, but whose properties are known. However, we will summarize the specific challenges of empirical data for each of the analyses presented later on in a section of pitfalls and issues. To generate a sequence of data points of the Lorenz-system dynamics, we use the *lorenz()*-function from the ‘nonlinearTseries’ package and store them in an arbitrary variable *lorData* (see Figures 2A–D for the resulting 3D Lorenz attractor and its component time-series):

lorData<−lorenz(time=seq(0,20,by=0.02),do.plot=F)

To generate the particular dynamics, the parameters sigma, rho, and beta have to be set, which we use here in their default-settings of the *lorenz()*-function (σ = 10; ρ = 28; β = 8/3). These three time-series will allow us to explore different levels of dynamics, e.g., between pairs of time-series (dyad-level dynamics) and triads of time-series (group-level dynamics in the Lorenz-system).

## Phase-Space Reconstruction and Parameter Estimation

As we said above, recurrence-based analyses are effectively generalized correlational analyses that provide information about different correlational characteristics of two or more time-series. As such, all of the analyses we present in the following can simply be run on the raw data of the time-series as they have been collected. Alternatively, these analyses can be used to compute correlations between the (reconstructed) phase-spaces dynamics of the time-series of interest. In a nutshell, using the logic of Takens (1981) theorem, it is possible to recover higher-order dynamics by the method of time delayed embedding of the time-series (Takens, 1981). If one suspects that there are more complicated dynamics behind the observed time-series, which is often the case when the measured variable is continuous, one needs to follow the embedding procedure outlined below to reconstruct the time-series’ phase-space, the first step of which is the estimation of the embedding parameters. In many other cases though, especially when time-series represent categorically coded behaviors (often the only kind of data available in the psychological and social sciences) or derivative measures (e.g., inter-event intervals), embedding may be unwarranted or unjustified from a theoretical point of view. In those case, we can still take advantage of the signal analysis capacities of recurrence based methods, by proceeding without any embedding of the observed time-series.

The estimation procedure for the embedding parameters is basically the same for all of the following recurrence-based analyses. The parameters are the embedding dimensions *m*, the delay *d*, the radius *r*, and the rescaling *norm*. The parameters *m* and *d* are necessary in order to estimate the correct dimensionality of the dynamics of a time-series. The parameter *r* is important to account for the fact that interval-scaled and empirically measured time-series usually contain noise, and never perfectly repeat themselves. Finally, the parameter *norm* standardize the analyzed time-series so that they are of comparable magnitudes. These parameter will later be used as inputs (in addition to the time-series data) for the recurrence analysis functions described below, which estimate coupling between time-series.

The embedding dimension *m* is an estimate of the dimensionality of the dynamics of the time-series – that is, how many latent variables, if any, compose the system whose dynamics are observed. For example, the Lorenz system presented in Figure 2A is 3-dimensional, but if we had only data from one of its dimensions available, we would need to estimate the number of the dimensions that we have not measured. The delay parameter *d* helps to recover latent dimensions using the method of time-delayed embedding that will be described below in more detail. Basically, *d* takes into account the sampling properties of a time-series, and helps to find points in the time-series that can be used to re-construct the missing dimensions most reliably. The radius parameter *r* effectively specifies the interval within which two values are counted as being recurrent, which is needed for interval-scaled data, that usually never repeats itself perfectly and contains measurement noise. The *norm* parameter is helpful for comparing different time-series with regard to their sequential structure, but that differ in the magnitude of their values. The *norm* parameter effectively brings the magnitude of the values of different time-series on the same scale, so that recurrence variables such as *%REC* and *%DET* are solely dependent on sequential properties, and not on differences in magnitude.

The necessity for selecting these parameters is easier to grasp from the perspective of basic RQA (one-variate recurrence analysis), where the aim is to quantify the recurrence structure of a single time-series, and not shared recurrences between multiple time-series (where these parameters are needed nevertheless). In that situation, we only have a 1-dimensional observable from a potentially multi-dimensional system – for example, the *x*-dimension from the Lorenz attractor (see Figure 2B). We could run recurrence-analysis just on the raw values of this time-series to describe its recurrence properties. However, since this 1-dimensional time-series comes from a 3-dimensional system of interacting and inter-dependent variables, its recurrence properties would be more accurately described if we could take the dynamics of the other two dimensions into account – and the procedure of phase-space reconstruction allows to do this.

In the case of the Lorenz system, we know that the actual system dynamics “live” in a 3-dimensional phase-space, and not in the 1-dimensional space of variable *x* taken alone. The phase-space is a multidimensional picture of the 1-dimensional time-series, obtained by plotting the values of the 1-dimensional time-series against each other at a certain delay. In a seminal paper, Takens (1981) showed that it is possible to reconstruct the missing information from the other, non-observed dimensions (in our case dimensions y and z from the Lorenz system). In particular, Takens showed that this missing information can be recovered via the method of time-delayed embedding (see also Sauer et al., 1991; Kennel et al., 1992).

**FIGURE 3:**
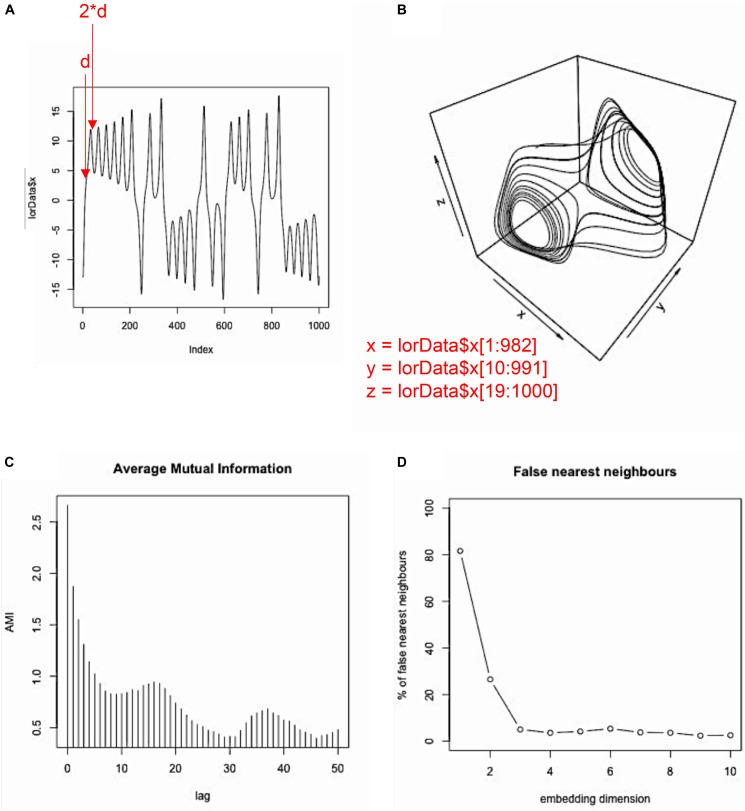
Phase-space reconstruction and estimation of embedding parameters. Example of phase-space reconstruction via the method of time-delayed embedding using the *x*-dimension of the Lorenz attractor. To that end, the original series **(A)** is plotted against itself at a certain delay *d* (*d* = 9 in this case; see panel **D** and the main text). This is done *m–*1 times, where *m–*1 is the number of additional surrogate dimensions needed in order to arrive at the correct dimensionality of the source system. Then, the original time-series and its two surrogates are plotted against each other, resulting in a reconstructed phase-space **(B)**, which is topologically isomorphic to the phase-space of the actual source system (see Figure 2A). The delay parameter *d* can be estimated using the average mutual information (AMI), where the first local minimum of that function provides a good estimate for *d* **(C)**, and the embedding parameter *m* can be estimated using the false nearest-neighbor (FNN) function, where the first local minimum (or the point at which the function becomes stable) provides a good estimate for *m* **(D)**.

The idea behind this so-called phase-space reconstruction with the method of time-delayed embedding is not complicated: If we only have a 1-dimensional time-series, and want to recover the time-series (or trajectory) in its multi-dimensional phase-space, we use *m-1* surrogate copies of the original time-series, sampled at lag *d* from each other (where d is the delay parameter) to derive the coordinates for every point of such trajectory. The resulting multi-dimensional scatterplot-like graph will approximate the topological dynamics of the actual multidimensional system (see Figures 3A,B for an example using the x-dimension of the Lorenz attractor), meaning that a properly embedded version of a 1-dimensional time-series in phase-space looks similar to the “true,” multidimensional trajectory.

As we will see, this logic is applicable to all of the analyses techniques presented in this paper. However, one can only safely rely on an established routine to do so for CRQA (and hence DCRP), which operate on the basis of individual time-series, while the issue of embedding is more complicated – and perhaps not always warranted – in the case of MdRQA (this will be discussed in the section “Multidimensional Recurrence Quantification Analysis” below).

The problem with empirical data is, that we usually do not know the dimensionality of the source system *a priori*. Hence, the dimensionality of the system has to be estimated, as well as the delay parameter that is needed to properly re-construct the systems phase-space. However, two methods have been proposed to estimate those parameters. To estimate the delay parameter, the average mutual information function (AMI) of the component time-series can be computed, and usually its first local minimum is taken to be a good estimate of the delay parameter *d* (for a detailed rationale of this choice see Abarbanel, 1996). Specifically, the first local minimum provides the lag where the time-series is most independent of itself, and embedding at this point will provide the most unique information for a new dimension in phase-space. In R, this can be done using the *mutual()*-function from the ‘tseriesChaos’ package, which we now want to apply to the *x*-dimension of the Lorenz system:

mutual(lorData$x,lag.max⁡=50)

Here, we chose to compute average mutual information for the first 50 lags of the time-series. As can be seen in Figure 3C, mutual information drops off, with a local minimum at about lag 9, after which mutual information increases again. Hence, 9 is our estimated value for the delay parameter *d*. Note that in our case, much less than 50 lags would have been needed to find the first local minimum. However, sometimes no clear local minimum is visible within a certain range of lags, in which case it is usually helpful to re-run the AMI function with a higher lag (i.e., set lag.max to a larger value).

Now, we know the delay that we need for the embedding process, *d* = 9. However, we do not yet know the dimensionality of our phase-space, that is how many times to apply the delay. The embedding dimension of a time-series can be estimated using the false-nearest-neighbor function (Kennel, et al., 1992). The idea behind this estimation procedure is, that if a time-series is not properly embedded with regard to the “true” dimensionality of its dynamics, values in the time-series are classified as similar/recurrent that should actually be treated as non-recurrent (i.e., they are “false” neighbors). As a rule of thumb, high levels of noise in the data tend to inflate the estimate of the embedding dimension *m*. Now, we want to use the *false.nearest()*-function (from the same R package) to estimate the embedding dimension *m*:

plot(false⋅nearest(lorData$x, m=10,d=9,t=0))

As input, the function takes *m* for the range of dimensions that we want to test, *d* for the delay parameter that we have estimated previously, and *t* for the so-called Theiler window (Marwan et al., 2007) that allows to specify the minimal (temporal) separation of neighbors. Effectively, *t* specifies the number of diagonals on a recurrence plot whose recurrence points are excluded for the computation of the recurrence measures, with *t* = 1 meaning that recurrence points at the main diagonal are excluded, *t* = 2 meaning that recurrence points at the two adjacent diagonals above and below the main diagonal are excluded, and so forth (see Marwan et al., 2007, p. 250, for a more extensive discussion). Similar to the AMI function, we look for a first local minimum and/or for a leveling-off in false-nearest-neighbors (see Figure 3D), where additional embedding dimensions do not appreciably change this number (similar to the scree-test in factor analysis – Cattell, 1966). The FNN-function is used because in a non-optimal reconstruction of the phase-space points (coordinates) can appear to be recurrent when in fact they are not, which would be visible in a higher dimensional phase space. Hence the name “false neighbors.” As can be seen in the plot, we observe only significant portions of false neighbors for embedding dimensions up to 2. Hence, 3 is our estimate for the embedding dimension parameter *m*, as it should be, because the Lorenz system actually is 3-dimensional (see Figure 2 and equation 1).

To sum up, plotting the data in *lorData$x* three times against each other with delay 9, will result in the reconstructed phase-space for the Lorenz system as in Figure 3B. That means, we take the original time-series, and plot it against itself two additional (i.e., *m-*1) times, where the second time the data points are shifted by 9 lags (i.e., 1^∗^*d*), starting with the 10th data point of the original time-series, and the third time the data points are shifted by 18 lags (i.e., 2^∗^*d*), starting with the 19th data point of the original time-series.

However, we are interested in recurrences of the same time-series in phase-space, and to quantify those, we can move from the phase-space representation to the recurrence plot representation of the time-series. In order to do so, we need to determine which values in the reconstructed phase-space are recurring. For nominal sequences and for simple deterministic systems that is no problem, but for time-series with complex dynamics and stochastic components and/or measurement noise, we need to threshold the phase-space first.

Thresholding means, that we define a range, the radius parameter *r*, within which we take two coordinates in phase-space to be recurrent – even if their values are not identical. However, a proper value for the radius parameter *r* cannot be estimated so easily. As said above, we do not need a radius for nominal sequences (or effectively we set the radius close to zero), because here we just consider repetitions of the same states or values. In fact, nominal sequences are the only kind of data that as something like a natural level of recurrence, because by selecting a tiny value for the radius, we allow only truly identical values to be counted as recurrent, with everything else being classified as non-recurrent. For all other kinds of data, recurrence (*%REC*) depends on the selection of *r*.

For highly deterministic time-series, small radii will suffice, while highly stochastic time-series or time-series with a strong noise component need big radii to count sufficient recurrences. In general, recommendations have been to set the radius *r* so that the resulting recurrence rate lies between *%REC* = 1 to 5% (see e.g., Webber and Zbilut, 2005), where it can be on the lower side of this estimate for time-series with a strong deterministic component, and on the upper side of this estimate for time-series with a strong stochastic component. However, very stochastic time-series (such as inter-event-times) might even warrant recurrence rates higher than that (Wallot et al., 2012). Radii are usually expressed in terms of their percentage of the norm parameter, or in terms of the standard deviation of the time-series when the input time-seris were standardized. Note that additional constraints are put on the selection of the radius in the context of comparing samples of data sets, which we will discuss at the very end of the paper.

Finally, a *norm* parameter needs to be set. The *norm* parameter is important for re-scaling phase-spaces of different time-series with regard to the magnitude of their values. This is, because we are interested in comparing different time-series in terms of their sequential properties, and differences in magnitude between two series could influence the estimation of their sequential similarities or differences (e.g., Shockley, 2005). Several *norms* have been proposed, for example normalizing the phase-space by the (average) Euclidean, Maximum, or Minimum distance in phase-space. Given circumstances, the choice of one *norm* can be an advantage over choosing another one, but the main point is to keep the *norm* parameter constant across all time-series that are analyzed to be compared across samples.

**FIGURE 4:**
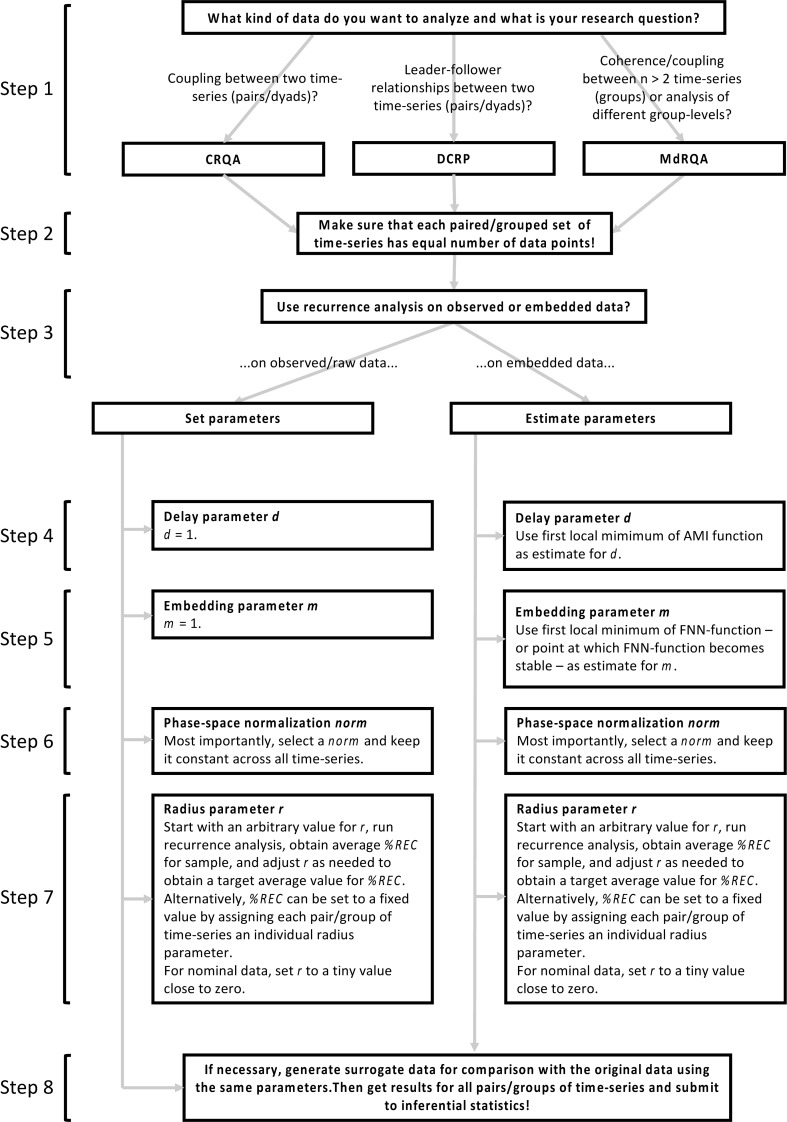
Main steps of selecting and conducting the analyses. Please note that this is only a gross overview over the main steps – for specific problems, best practice, or tuning of parameters, please consult the respective sections on these steps in this article.

Conducting estimation of the delay and embedding dimension parameters using the *mutual()*- and *false.nearest()*-functions for our three time-series from the Lorenz-system leads to the results presented in Table 2, which we will need as inputs for the analyses that we are going to introduce in the next sections. Figure 4 summarizes the main steps of selecting and conducting the analyses. For details and solution for specific problems, consult the relevant sections of this article.

**Table 2:** Delay (*d*) and embedding dimensions (*m*) estimated for the six time-series.

Time-series	*d*	*M*
*lorData$x*	9	3
*lorData$y*	8	4
*lorData$z*	8	4


*The above values were simply selected according to a strict application of the first-local-minimum criterion. In case of doubt, one can test whether other reasonable delays lead to markedly different outcomes. Note that the *false.nearest()*-function in R suggests that *y*- and *z*-coordinate data come from a 4-dimensional system, even though we know that the actual system is 3-dimensional. However, such minor differences usually do not matter for the results. In fact, building a recurrence plot on a 3 vs. 4-dimensional phase-space will leads to very similar results, and as a rule of thumb, it is advisable to slightly over-embed (i.e., pick an embedding dimension slightly higher than the estimate) when in doubt.*

## Pitfalls and Issues

The presence of noise in a time-series can inflate estimates of embedding dimension using the false-nearest-neighbor algorithm. On the one hand, this is not very problematic, because moderately over-embedding a time-series (i.e., picking higher values for *m* than the actual dimensionality of the dynamics of the system from which the time-series was recorded) usually does not impact the results of recurrence-based measures very much, and the analyses are generally robust across a certain range of parameter settings. Alternatively, one can carefully smooth the time-series before estimation embedding dimension. However, even in this case, one might still want to do the first analyses on the unsmoothed data, because correlated noise properties can actually contain information about the time-series dynamics, and recurrence-based analysis can harness such information.

Finally, there are data sets for which no clear local minimum in either the AMI and/or the false-nearest-neighbor function are observed. In this case, one can pick values for *d* and *m* when the functions level out. Often, however, this means having relatively high values for *d* and *m*, and because large embedding parameters actually reduce the number of data points available for analysis, one needs time-series with sufficiently many data points. Alternatively, lower values for *d* and *m* can be picked by investigating the function for instances for “substantial decreases” instead of straight local minima or leveling. Of course, selecting parameters on this basis will increase the subjective component in parameter setting, and one way to deal with this would be an exploration of the parameter space. Here, one selects a handful of different parameter combination based on the different criteria presented above, and check whether the results of the analysis are invariant or at least similar across these. In general, picking smaller values for *d* and *m* results in more and/or “false” recurrences, while picking bigger values of *d* and *m* results in fewer recurrences. However, picking slightly higher values, particularly for *m*, will still result in reliable and correct classification of recurrences.

## Cross-Recurrence Quantification Analysis (CRQA)

**FIGURE 5:**
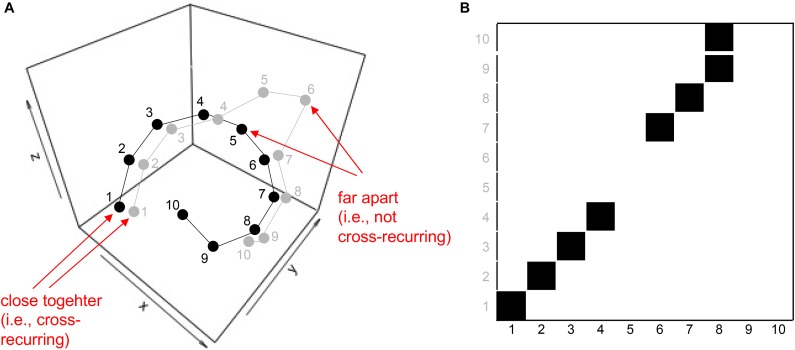
Schematic of embedding two time-series in a single phase-space **(A)**, and charting cross-recurrence between coordinates of the two time-series in a cross-recurrence plot **(B)**. Note that the indices on the *x*- and *y*-coordinates in panel **B** here do not display the values of the associated time-series, but the order in which these values appear in the time-series (i.e., the gray 10 is the 10th coordinate of the gray coordinate-series in the phase-space in panel **A**).

Cross-recurrence quantification analysis is a bivariate correlation technique. Instead of quantifying recurrences within a time-series (as RQA does), CRQA quantifies cross-recurrences between two time-series. This bivariate extension of RQA is conceptually pretty straightforward: Instead of embedding a single time-series in a phase-space (see Figure 3), two time-series are embedded in the same phase-space (see schematic in Figure 5A). Note that the numbers next to the two time-series indicate the order in which the data points (coordinates) have been measured, not the values of the data points! The data are arbitrary, and are meant to illustrate how embedded time-series in a phase-space are used to construct a cross-recurrence plot (CRP).

Now, the cross-recurrence plot is constructed by charting instances where coordinates of the two time-series occur close to each other in the phase-space – again within a given radius size *r* (Figure 5B). This cross-recurrence plot can now be quantified in the same fashion as the simple recurrence plot (see Table 1). Note, however, that the cross-recurrence plot for two different time-series does not necessarily possess recurrences at the central diagonal, and is not symmetric about the diagonal anymore. We can make use of this asymmetry to assess lead-follower relationship or lags in coupling when examining the diagonal cross-recurrence profile, which is done in the next section.

Also note, that the cross-recurrence plot, similar to the recurrence plot, charts recurrences at all possible lags for the two time-series. Hence, the cross-recurrence measures do not reflect coupling at a specific lag or within a specific time-interval only.

The sequence of steps for conducting CRQA is very similar to that of RQA: First, the AMI and the false-nearest-neighbor function are used to estimate the delay and embedding dimension parameters for each time-series separately. If the time-series exhibit the same delay and dimension, then those parameters are used for CRQA. If the time-series differ in delay and dimension, then one can use either the average values for each parameter (rounded to the next integer), or the highest values for each parameter – delay *d* and embedding dimension *m*. Again, as a rule of thumb, over-embedding is less problematic compared to under-embedding (Webber and Zbilut, 2005) – given enough data points, of course. Then, appropriate *norm* and radius parameters *r* need to be selected, and usually the data are normalized beforehand (e.g., *z*-scored, or transformed to unit interval), to ensure that CRQA measures are really based on sequential similarity of the two time-series, and not differences in magnitude (Shockley, 2005).

## Running the Analysis in *R*

As we have already estimated embedding parameters *d* and *m* for our time-series (see Table 2), we can skip this step and directly proceed to the application of the *crqa()*-function from the ‘crqa’ package in R. To begin with, we will perform CRQA on the same time-series – that is, on *lorData$z* and *lorData$z* – so two time-series with the same values (see Box 1).

**Box 1. Running CRQA.**

crqa_results_ab <– crqa(ts1 = lorData$z, ts2 = lorData$z, delay = 9, embed = 3, rescale = 2, radius = 20, normalize = 2, mindiagline = 2, minvertline = 2, tw = 0, whiteline = FALSE, recpt = FALSE, side = “both”) # running crqa

image(crqa_results_ab$RP) # cross-recurrence plot

print(crqa_results_ab[1:9]) # crqa results

The *crqa()*-function has several arguments to be defined: *ts1* and *ts2* are the two time-series that should be analyzed; *delay* is the delay parameter *d*, *embed* is the embedding dimension *m*, *rescale* is the *norm* parameter used to rescale the phase-space (2 equals maximum rescaling), and *radius* is the radius parameter *r*. The parameter *normalize* is an option to normalize the data beforehand (2 equals *z*-scoring). The parameters *mindiagline* and *minvertline* allow the user to select the minimum number of diagonally and vertically adjacent points that should go into the calculation of the recurrence metrics (the default being 2, i.e., any point that is not isolated is counted as a line). *tw* is the Theiler window (see section “Parameter Estimation”). Interested readers are referred to the article by Coco and Dale (2014), as well as the description of the *crqa()*-function in the ‘crqa’ package.

**FIGURE 6:**
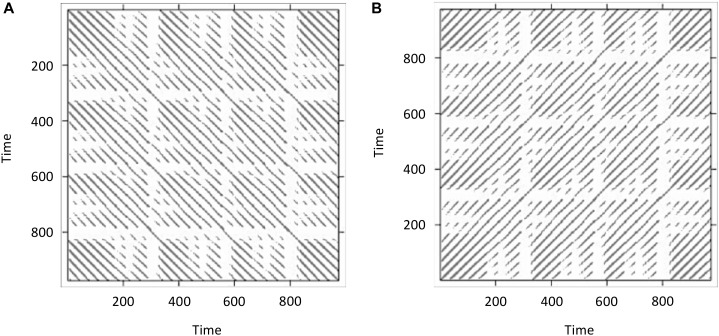
Doing recurrence analysis using cross-recurrence: Display of cross-recurrence plots resulting when the same time-series data (*lorData$z*) is added for both inputs as plotted directly from the output of the *crqa()*-function **(A)**. However, recurrence plots are conventionally oriented so that time at lag0 runs along the main diagonal from the lower-left to the upper-right. Hence, the resulting cross-recurrence plot need to be rotated by 90° **(B)**.

Figure 6A displays the resulting cross-recurrence plot. First of all, notice that when plotting the resulting recurrence plots using the *image()*-function in R, the recurrence plots are rotated in conventional matrix orientation, where the smallest values of the indices begin at the upper-left. Hence, time in this plot now runs from the upper left to the lower right (instead of the conventionally used direction from lower left to upper right – which we have used in Figures 1, 5B). This is important to pay attention to, because in different publications, different ways to display recurrence plots are chosen in this regard. This can be remediated by transposing the matrix with the *t()*-function on the reversed columns of the matrix before plotting, resulting in the conventional orientation where time at lag0 now runs again from the lower-left to the upper-right (see Figure 6B):

$$RP<--crqa\_results\_ab\$RP\#\text{store cross-recurrence plot in variable RP image}\left(t\left(RP\left[,ncol(RP):1\right]\right)\right)\#\text{rotate matrix by }90^{{}^{\circ}}\,\;and\,\;plot$$

What we have done here was effectively to compute RQA (i.e., an analysis of the auto-recurrence properties of a single time-series) using CRQA, because we entered the same time-series twice. Hence, we observe the central diagonal on the plot, and see that the plot is symmetrical about this diagonal. If these were two independently collected time-series, it would mean that they are perfectly synchronized, exhibiting the same dynamics at the same time.

**FIGURE 7:**
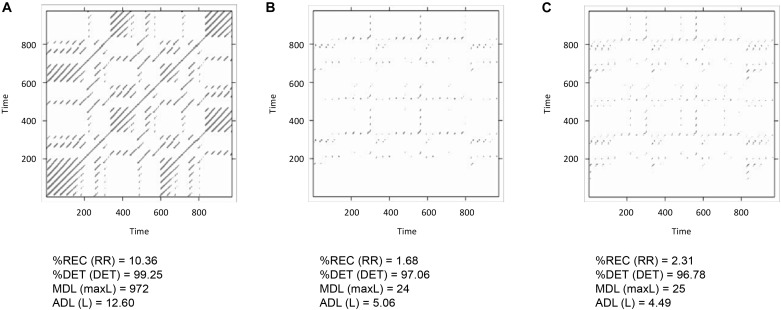
Display of cross-recurrence plots and resulting CRQA measures the time-series pairs *lorData$x* with *lorData$y* **(A)**, *lorData$x* with *lorData$z* **(B)**, and *lorData$y* with *lorData$z* **(C)**.

Finally, we will run three pairwise analyses of the time-series *lorData$x*, *lorData$y*, and *lorData$z* (see Box 2). The results are displayed in Figure 7. Comparing the cross-recurrence plots of the three pairings, we see that they do not quite exhibit the same patterns, but all of them seem to consist of relatively grouped or connected recurrent points. Examining the CRQA results corroborates this: All three pairs have high values of cross-*%DET*, meaning that cross-recurrences do not occur in isolation, but in connected groups. Still, for some of the CRQA outcome measures, the numbers differ substantially between pairs. This shows, that the three dimensions of the Lorenz-system do not possess that same intrinsic dynamics, and are not all coupled together in the same way. Here, we will see that it can be useful to examine multiple time-series together as in multidimensional recurrence analysis, which will introduce in the section after the next one.

**Box 2. Running all pairwise CRQAs for the three dimensions of the Lorenz system.**

*crqa_results_xy <– crqa(ts1 = lorData$x, ts2 = lorData$y, delay = 9, embed = 4, rescale = 2, radius = 20, normalize = 2, mindiagline = 2, minvertline = 2, tw = 0, whiteline = FALSE, recpt = FALSE, side = “both”)* # running crqa between the x and y dimensions of the Lorenz system

*crqa_results_xz <– crqa(ts1 = lorData$x, ts2 = lorData$z, delay = 9, embed = 4, rescale = 2, radius = 20, normalize = 2, mindiagline = 2, minvertline = 2, tw = 0, whiteline = FALSE, recpt = FALSE, side = “both”)* # running crqa between the x and z dimensions of the Lorenz system

*crqa_results_yz <– crqa(ts1 = lorData$y, ts2 = lorData$z, delay = 9, embed = 4, rescale = 2, radius = 20, normalize = 2, mindiagline = 2, minvertline = 2, tw = 0, whiteline = FALSE, recpt = FALSE, side = “both”)* # running crqa between the y and z dimensions of the Lorenz system

## Pitfalls and Issues

As all correlational techniques, CRQA (and also DCRP and MdRQA) need two matched time-series. If time-stamps of the recordings are synchronized, and time-series are sampled continuously, this is no problem. However, if the time-series are sampled as inter-event-times (such as recording heart rate as beat-to-beat intervals, response times, or iterated maps), two synchronously recorded time-series can be of different length. This can be fixed by transforming the time-series into a pseudo-continuously sampled series using time-normalization methods. However, the fluctuations in the original inter-event series can get smoothed-out in the process, which is undesirable, because these fluctuations might contain actual information that recurrence-based techniques can uncover. Hence, when transforming inter-event to pseudo-continuous time-series for the purposes of subjecting them to CRQA, one should pick a high re-sampling rate at a relatively small window, because this will result in higher accuracy of CRQA results (Wallot et al., 2013).

Another issue concerns the question of whether or how to normalize the individual time-series before submitting them to CRQA. If the values of the time-series have similar distribution and magnitude of values, then normalization should not make much of a difference. If they do, then it is helpful to normalize each time-series to ensure that the CRQA results are really a property of the sequential order of data points in the time-series, and not due to differences in magnitude. However, extreme outliers of extreme differences in distributions (especially if one of them possesses a strong heavy tail) may result in the normalization procedure to introduce uncertainty into the CRQA results instead of reducing it. Recurrence-based procedures themselves are extremely robust against outliers, but this is not true for certain normalization procedure, since, for example, *z*-scoring a time-series with one or few extreme outliers results in “squeezing” the remaining mass of the data points together. Here, either removing and replacing individual data points can be warranted (if they are very few), or otherwise normalizing data based on percentiles. In the particular case where the two time-series have similar distributions and magnitudes of values, but one of them contains outliers, an option would be not to normalize the data before the analysis but center the variables (see e.g., Shockley, 2005, p. 163).

However, even under those circumstances, CRQA is relatively robust, you can try this out by entering two time-series that differ in magnitude into the analysis with and without normalization. Given some adjustment of the radius parameter to yield some sufficient level or cross-recurrence points, the cross-recurrence plot should still look similar under both circumstances, and also the other CRQA results should be in the same ball park.

Finally, sometimes it is hard to evaluate whether CRQA results are indicative of substantial coupling between time-series, or how strong this coupling is. After all, by choosing a sufficiently high value for the radius parameter *r*, one can even make two random number sequences yield cross-recurrences (even though they look like homogenously distributed isolated cross-recurrence points). There are two procedures that have been suggested to obtain base-line measures of cross-recurrences. First of all, one can randomly shuffle both time-series (to do this, use for example the R function *sample()*, which returns shuffled values of its input variable) and then do CRQA with the same parameters of the original time-series. This assesses, how far the two time-series exhibits coupling above chance. Another way is to calculate and average the individual recurrence properties for each time-series, and use this number as a reference point. This tells one how well the two time-series correlate, given that each of the two component time-series possess some dynamic structure that might influence the CRQA results. Here, however, keep in mind that recurrence analysis results of individual time-series – everything else equal – are numerically higher than the cross-recurrence results. So, the question is, how well the CRQA results approach the average recurrence properties of the individual time-series, which do set some upper limit on cross-recurrence. If one deals with multiple samples (e.g., multiple dyads that perform different tasks), one can also create false-pairs to evaluate coupling strength, and we will come back to this procedure at the end when addressing pitfalls and issues of DCRP.

## Diagonal Cross-Recurrence Profile (DCRP) Analysis

After introducing cross-recurrence plots, we can now consider DCRP analysis (Richardson and Dale, 2005; Dale et al., 2011a,b) as a kind of follow-up analysis of the cross-recurrence plot. Instead of quantifying the whole cross-recurrence plot of two time-series, we restrict ourselves to only one measure (*%REC*) and to a limited band around the Line of Synchrony (LoS), the main diagonal of the plot (which usually spans the plot from the lower left corner to the upper right one). In principle other diagonal recurrence variables could be computed as well (such as *%DET* or *ADL* for each diagonal), but applications of these measures in DCRP-analysis have been sparse so far.

As we already mentioned in previous sections, the main diagonal in a cross-recurrence plot is special, because it captures recurrences between the two time-series at the very same time-stamp (lag 0), hence the name LoS. So, if the two time-series have equal or similar values (i.e., within the selected radius) at the same time-stamp, a recurrence point will be charted on the LoS, and this is why, for example, in RQA the main diagonal is always filled with recurrence points: a time-series is necessarily equal to itself at lag0.

When the time-series come from different, possibly coupled systems, the main diagonal will not always contain recurrences and the cross-recurrences will be asymmetrical distributed around it. The distribution of recurrence points around the diagonal could then indicate that some values from one time-series are followed by the same values in the other – if one of them leads and the other follows with a given lag or range of lags.

**FIGURE 8:**
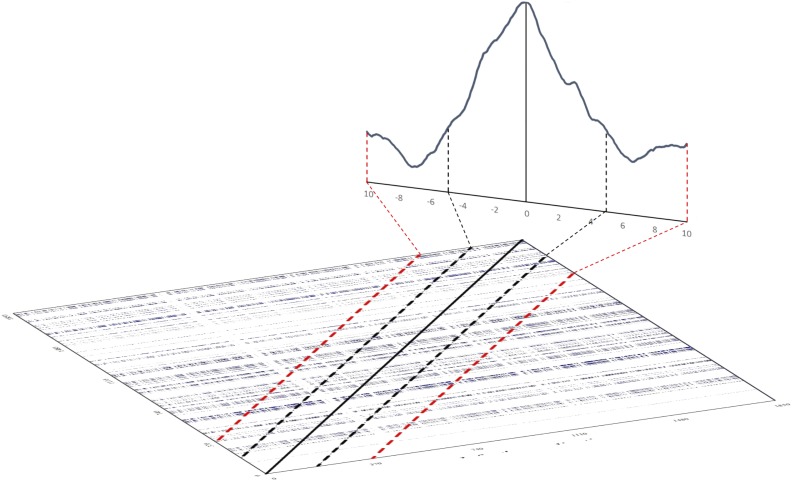
Illustration of how to compute a diagonal recurrence profile from a cross-recurrence plot. The solid black line on the tilted cross-recurrence plot marks the LoS. The dotted black and red lines show the width of lags ±5 and ±10 around the LoS, respectively. The line graph in the back of the figure illustrates the summation of recurrence points for the lags – the diagonal cross-recurrent profile.

We can then compute the relative amount of recurrence points, i.e., *%REC*, falling on a limitited set of diagonals (i.e., a limited number of lags) in a window centered at lag 0, which corresponds to the LoS. In this way, we can consider how recurrences are distributed across the set of lags chosen. The DCRPs are then the graphical representation of the amount of recurrence as a function of the lags. Figure 8 illustrates the relationship between the cross-recurrence plot and the diagonal recurrence profile that can be computed around the LoS.

## Running the Analysis in *R*

The core function to perform DCRP in R is to be found in the package ‘crqa’ (Coco and Dale, 2014), and is called *drpdfromts()*. The main focus of the whole package is on cross-recurrence analysis, which is an advancement relative to other non-linear time-series packages in R, but its application is also slightly biased toward categorical (nominal) time-series. Although, the *drpdfromts()-function* provides the option of choosing continuous input time-series, it is not possible to overrule the predefined settings of embedding (*m*) and delay (*d*) for the categorical case (*m = d = 1*). So, even though we already estimated that the adequate embedding for our data is *m* = 4 and the adequate value of delay is *d* = 9, we will have to perform our DCRP analysis on those data with no embedding and delay (i.e., both equal to 1). But first it is necessary to normalize the data to make the fluctuations comparable in phase space, since this option will also not be given by the *drpdfromts()*-function. Then, we can run the main analysis on the normalized data and save the results in appropriately named objects (see Box 3).

**Box 3. Data preparation and running DCRP on the three possible pairing of the dimensions of the Lorenz system.**

lorDataz <– as.data.frame(lorData[2:4]) # transform the Lorenz data in a data.frame

lorDataz <– as.data.frame(scale(lorDataz)) # normalize the three variables keeping the data.frame format

dcrp_results_xy <– drpdfromts(t1 = lorDataz$x, t2 = lorDataz$y, ws = 20, datatype = “continuous”, radius = 0.05) # run DCRP on the x and y dimension of the Lorenz system

dcrp_results_xz <– drpdfromts(t1 = lorDataz$x, t2 = lorDataz$z, ws = 20, datatype = “continuous”, radius = 0.05) # run DCRP on the x and z dimension of the Lorenz system

dcrp_results_yz <– drpdfromts(t1 = lorDataz$y, t2 = lorDataz$z, ws = 20, datatype = “continuous”, radius = 0.05) # run DCRP on the y and z dimension of the Lorenz system

The arguments we need to provide to the function are the two time-series we are analyzing (*t1* and *t2*); a value for the window size or the number of lags to compute around the LoS (*ws*; this value is the number lags on each side of the LoS, which in this example gives a total number 41 lags – 20 on each side of the LoS, plus the LoS itself, which is lag0); Hence, by setting the window size, one specifies the time interval within which one wants to compute time-lagged recurrences. Furthermore, one needs to set the type of the data (“categorical” or “continuous,” even though this choice is of no practical implications in the current version of the function); and finally the value of the radius, expressed in the same units as the time-series (*z*-scores in this case). The particular choice of radius in this case was guided by the principle of keeping the *%REC* value in the resulting recurrence plot at a relatively low level, here about 2.5%.

The following code will create the three plots of the profiles for the above analyses.

plot(−20:20,dcrp_results_xy$profile,type=“l″,xlab=“Lag″,ylab=“%REC″)

plot(−20:20,dcrp_results_xz$profile,type=“l″,xlab=“Lag″,ylab=“%REC″)

plot(−20:20,dcrp_results_yz$profile,type=“l″,xlab=“Lag″,ylab=“%REC″)

**FIGURE 9:**
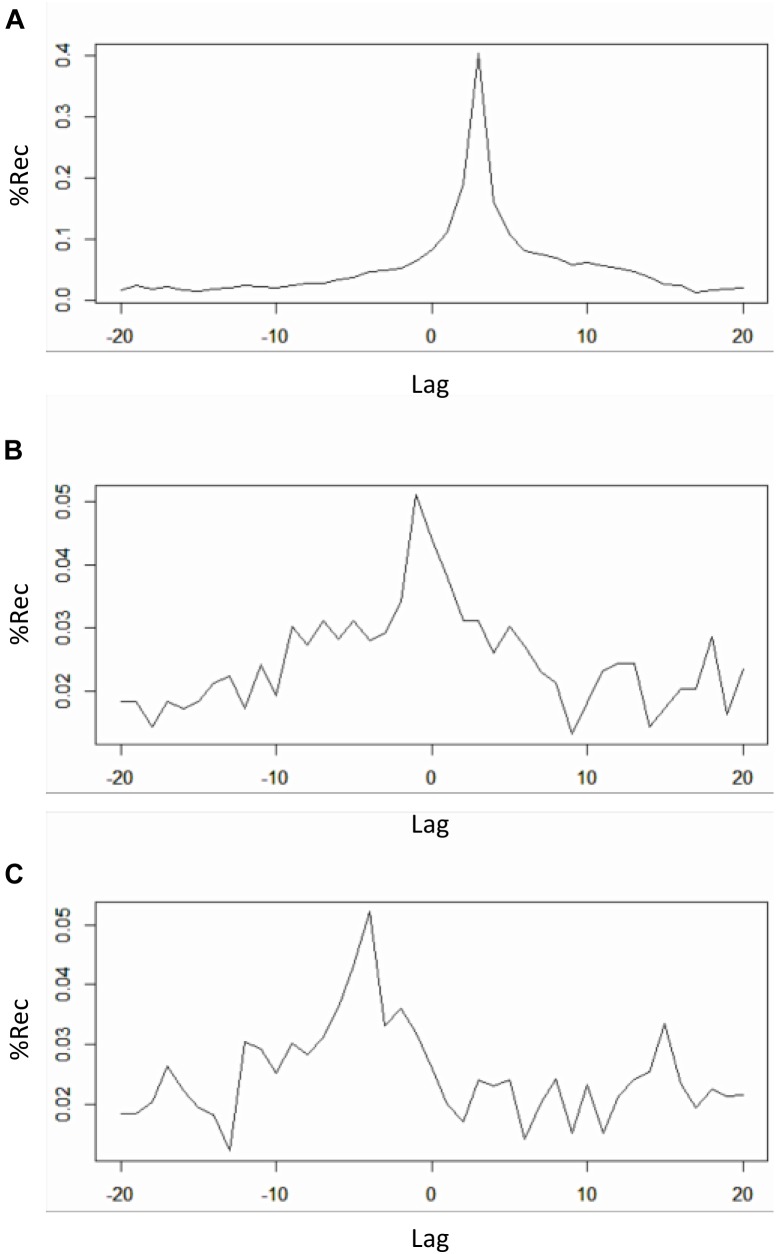
Recurrence profiles of the pairwise cross-recurrence analyses of the 1-dimensional *x-y-z* time-series from the Lorenz system. Plot of *drcp_xy$profile* (negative lags = *y* lagging behind) **(A)**. Plot of *drcp_xz$profile* (negative lags = *z* lagging behind) **(B)**. Plot of *drcp_yz$profile* (negative lags = *z* lagging behind) **(C)**.

Figure 9 shows the profiles for the three bivariate analyses. Table 3 shows the results outputted by the function, that is the value of maximal recurrence and at which lag it specifically occurs (Note: this value is to be read as the index in the vector going from -ws to +ws, and hence it needs to be translated in the appropriate, real time lag which is application specific).

**Table 3:** Output of the *drpdfromts()*-function.

	dcrp_results_xy	dcrp_results_xz	dcrp_results_yz
maxrec	0.403	0.051	0.0522
maxlag	24	20	17


*Apart from the profiles (shown in Figure 9), the function *drpdfromts()* usually provides two other values as output, the maxrec, which is the value of recurrence at the point of maximum in the profile, and the maxlag, or the index lag of the point of maximum. In the table results for these two values are presented for the analyses run.*

When eyeballing the profiles, we search for peaks emerging from a more uniformly distributed recurrence line. In this case a clear peak is evident in all the panels of Figure 9. In the cross-recurrence analysis of dimensions *x* and *y* of the Lorenz system (Figure 9A) the peak reaches its maximum value at about lag +3 (the 24th value of the profile’s vector, see Table 3) which means – given the order we entered the two time-series in the *drpdfromts()*-function – that *x* is consistently lagging behind y by 3 time steps. In other words, values of the y time-series recur with a lag of 3 within the *x* time-series (look also at Figures 2B,C). In the other plots, we also see a clear peak at lag -1 for time-series *x* and *z* (Figure 9B), meaning these two time-series are almost perfectly synchronized in their fluctuations (see also Figures 2B–D), while the peak is at a more negative lag (-4) for time-series *y* and *z* (Figure 9C), meaning in this case that *z* is lagging behind y of four time steps. In both cases the amount of maximum recurrence is considerably lower than in Figure 9A. which is understandable given the different type of oscillation of time-series *z* compared to the other two (see Figures 2B–D).

**FIGURE 10:**
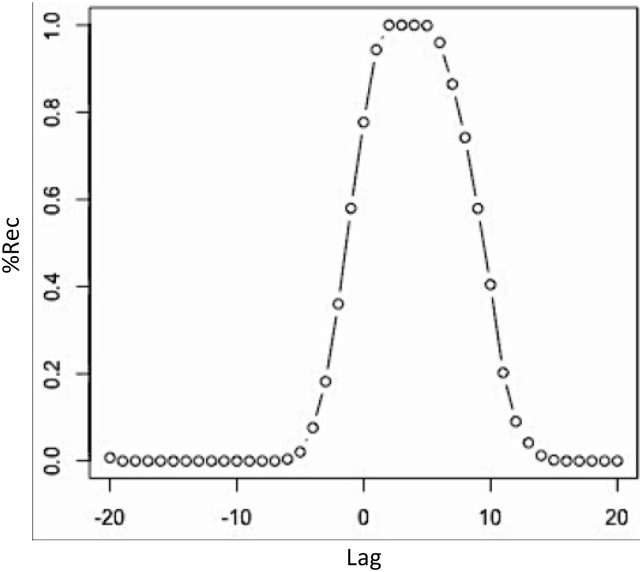
Recurrence profiles of the pairwise cross-recurrence analyses of the properly embedded time-series from the Lorenz system. Plot of the diagonal cross-recurrence profile of *lorData$x* and *lorData$y*, with the percentage of recurrence points plotted on the *y*-axis, and the number of lags around the LoS on the *x*-axis.

If we want to compute the recurrence profiles for embedded time-series, we will have to compute the cross-recurrence plot with proper embedding first, and then use the following routine to compute the profile from that plot (see Box 4). Here, we use the *crqa()*-function with the estimated embedding parameters and calculate the recurrence profile around ±20 lags around the LoS. The results are plotted in Figure 10. Comparing Figure 9A with Figure 10, we see that both, the non-embedded and the embedded version suggest a lag of +3, even though we see a maximum of recurrence points across multiple lags in Figure 10. This, however, is also a function of the comparatively higher value that we picked for the radius parameter.

**Box 4. Computing DCRP for embedded data.**

crqa_results_xy <– crqa(ts1 = lorData$x, ts2 = lorData$y, delay = 9, embed = 4, rescale = 2, radius = 20, normalize = 2, mindiagline = 2, minvertline = 2, tw = 0, whiteline = FALSE, recpt = FALSE, side = “both”) # compute cross-recurrence plot

diagLine <– split(crqa_results_xy$RP, row(crqa_results_xy$RP) - col(crqa_results_xy$RP)) # sort CRP into diagonal lines

lags <– 20 # chose number of lags around LoS

recLag <– 0 # create variable to store recurrences at specific lags

for (i in seq(lags+round(length(diagLine)/2), -lags+round(length(diagLine)/2))) { tempDiagLine <– unlist(diagLine[i])

recLag <– append(recLag, sum(tempDiagLine)/length(tempDiagLine)) } # loop through diagonal lines and calculate recurrences at each diagonale

plot(seq(-lags, lags), recLag[2:(lags^∗^2+2)], type = ‘b’) # plot DCRP profile

## Pitfalls and Issues

As already pointed out, the function available in R to compute DRCPs was specifically intended to analyze categorical time-series. Hence, when time-series are categorical, some cross-recurrence analysis parameters are set by default, usually *m* = 1, *d* = 1, and *r* = 0, when using function *drpdfromts()*. It is important to be aware of this limitation when running our analysis or alternatively use the code provided in Box 4 for the computation of continuous and embedded data.

A few problems specific to this analysis have to do with the evaluation of the profiles. The most important one concerns the relevance of the points of maxima (and minima) in the profile, indicating a concentration of recurrence (or some particularly low level of it) across a certain range of lags. In general, a maximum is always present in the profile we obtain, so the question is whether the amount of recurrence at this lag (or set of lags) is indicative of a real lag-dependent coordination in the two time-series or whether it is no different from the amount of recurrence we see at other lags. In other words, we need a reference point against which to compare the peaks and valleys in the profile.

This reference point has usually been found in a recurrence baseline, which is an additional profile we can plot alongside the original one. A few different methods have been proposed for the generation of baseline profiles. One proposal is the creation of so-call false-pairs, extracting the diagonal-wise profiles from a surrogate pair of time-series which come from the same experimental condition, but different participants. This would effectively eliminate the fine-grained time dependent coordination we would expect in coupled systems without disrupting the actual, ordered nature of the time-series themselves. Another more radical proposal is to eliminate the ordered, sequential nature of the time-series by shuffling them randomly and then run cross-recurrence analysis on them, maybe even repeating the process several times to finally take the averaged profile and confidence intervals from these repetitions. In the case of categorical time-series, a similar procedure would also be to shuffle the time-series without ‘breaking’ the chunks of equally coded events in which the time-series is organized. As of now, it seems good practice to compute both kinds of baslines in order to evaluate specific parts of the DCRP. Moreover, there are other possibilities to create surrogate series that retain specific moments of the data and remove others, and can be used to test for whether the presence of absence of these moments are driving the observed effects (such as the iterative amplitude-adjusted fourier-transformation, IAAFT – Schreiber and Schmitz, 1996). However, one needs to carefully check what moments such surrogates are manipulating in order to arrive at a proper interpretation of the results.

We need also to underline the fact that the coupling in the two time-series ought to be rather strongly time-locked, for it to appear as a clear peak in the DRCP. In fact, if the time delay between similar states in the two time-series is highly variable, the recurrence will spread across several diagonal lines close to the LoS and hence we could possibly fail to see a peak at any definite lag. This might, however, be itself exactly the kind of information that is of interest.

## Multidimensional Recurrence Quantification Analysis (MdRQA)

Multidimensional recurrence quantification analysis is a multivariate extension of simple RQA for multidimensional time-series, and can be used to analyze the joint dynamics of groups (*n* > 2) of participants (Wallot et al., 2016b). The underlying idea of MdRQA is simple: Instead of using a single 1-dimensional time-series that is embedded in phase-space, one uses multiple recorded time-series that are embedded in a phase-space. However, in contrast to CRQA, it is not that each of these time-series is embedded separately into one phase-space, but each time-series actually provides one (or multiple – if the time-series is further embedded via time-delayed copies) dimension of that phase-space.

**FIGURE 11:**
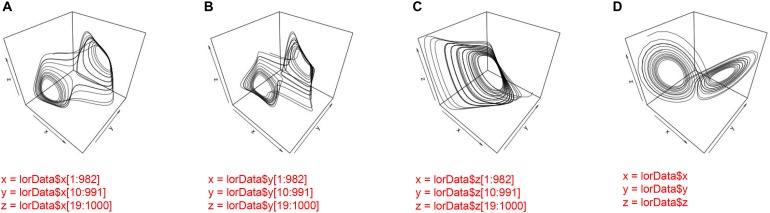
Phase-space reconstruction through 3-D embedding of the individual time-series of the Lorenz-system **(A–C)** and the phase-space portrait of the actual 3-dimensional Lorenz-system **(D)**.

Consider the data from the Lorenz-system that we have generated. We know that the system is 3-dimensional, and we have a time-series that corresponds to the variation on each dimension over time. However, we also know from our analysis of these three time-series in CRQA that not all of the three possible pairings yield the same results. Hence, correlating all dyadic time-series does not properly reflect the system-level dynamics of the Lorenz-system. Figure 11 displays the three phase-spaces reconstructed from each dimension of the Lorenz system and the 3-dimensional original.

No matter whether one interprets the three variables as a multidimensional behavior of one system, or the coupled behavior of three individual systems, MdRQA makes it possible to quantify such higher-level dynamics properly, by taking the phase-space of multiple measures time-series as the point of departure. Also, it allows to quantify the dynamics at different grouping levels – for example a group of 4 participants which act together in a task and where each participant provides one observable can be analyzed in terms of six possible dyads (MdRQA2) within this group, four possible triads (MdRQA3) within the group, and one group-level containing all four members (MdRQA4).

However, even though MdRQA offers new possibility for the analysis of multivariate time-series, is also has certain limitations. If MdRQA is run on un-embedded multidimensional time-series, certain dynamics of the system might not be properly captured if not all dimensions of the system are adequately represented as dimensions of the multivariate time-series. Particularly with empirical data, the problem is that – in contrast to the Lorenz system example we are using here – we usually do not know the dimensionally of the data *a priori*. Of course, data can be embedded using MdRQA as well, but the embedding parameters have to be estimated on the individual component dimensions of the multidimensional time-series and such an estimation might not properly represent the actual parameters of the multidimensional time-series (see section “Issues and Pitfalls”).

## Running the Analysis in *R*

To run the analysis in R, you need to copy-paste the *mdrqa()*-function from the Supplementary Materials to this paper into your workspace (or download it from a repository - see the link in the Supplementary Materials). Now, we want to use the three time-series *lorData$x*, *lorData$y*, and *lorData$z* to run a MdRQA3, that is an analysis with three time-series. This also means that the underlying phase-space is at least 3-dimensional. Of course, on top of that we might have to embed the time-series via the method of time-delayed embedding. However, checking the individual estimates of *m* for the three time-series (Table 2), it seems that all time-series are 3- or 4-dimensional. Hence, we decide not to embed anymore, because minimum dimensionality of phase-space (i.e., 3) is already pretty close to those estimates.

If the estimates of individual time-series dimensionality through the false-nearest-neighbor function would be substantially higher, for example 6, then we would additionally embed the time-series a single time at their estimated average (or maximum) delay. Because each of the three time-series already contributes one dimension to the phase-space, embedding this 3-dimensional time-series once would already yields a 6-dimensional phase-space.

To use the three time-series without further embedding, we call the *mdrqa()*-function (see Box 5). The data for the *mdrqa()*-function needs to be entered as a single matrix with each time-series being a separate column in the matrix and all the data points in rows. Hence, the *as.matrix()*-function and the *cbind()*-function are used to convert the three time-series into a matrix with three columns. *emb* is the embedding dimension, and since we do not want to embed the time-series further, we set *emb* = 1. *del* is the delay parameter, *norm* the parameter for phase-space normalization, and *rad* the radius parameter just as in the analyses above.

**Box 5. Computing MdRQA3 for the full 3-dimensional Lorenz system.**

mdrqa3_results <– mdrqa(data = as.matrix(cbind(lorData$x, lorData$y, lorData$z)), emb = 1, del = 1, norm = ‘euc’, rad = 0.2) # run MdRQA3 on the x, y and z dimensions of the Lorenz system

image(mdrqa3_results$RP) # show recurrence plot

**FIGURE 12:**
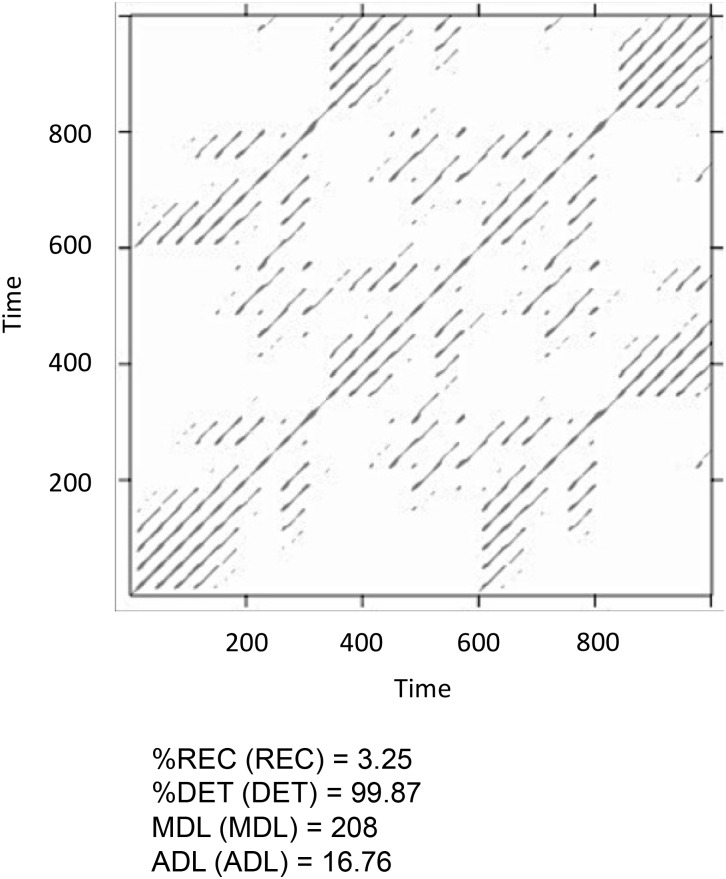
Recurrence plot and results of the MdRQA3 analysis for the time-series *lorData$x*, *lorData$y*, and *lorData$z*.

Figure 12 shows the results of the MdRQA3. Because MdRQA treats the time-series as a single multidimensional series, the resulting recurrence plot is symmetric. Similar to CRQA, we face the problem that the absolute values of the results are dependent on the radius parameter, and that we do not know how to evaluate them in terms of whether there is strong or weak coupling between the three time-series. As suggested in the Section “Cross-Recurrence Quantification Analysis,” one could perform an additional analysis with all time-series shuffled, or set the results in relation to the individual series’ RQA results. Note, however, that higher values for MdRQA are not necessarily an index of superior group coordination (Wallot et al., 2016a,b). Sometimes, looser coupling on the group level can be beneficial for task performance than stronger coupling, implying that lower MdRQA results are positively correlated and more predictive of group performance (see also Abney et al., 2015; Vink et al., 2017). In other words this is an interesting, open empirical question that MdRQA analysis can help to address.

Next, we could investigate whether there are differences on the dyadic level that could be of interest – for example, stronger coupling between some of the dimensions of the Lorenz-system compared to others. Translated into group data analysis, such an analysis allows to investigate whether all members of a group are equally interacting with each other or whether there are some preferred dyads within a group that tend to couple their behavior more (or less) with each other than with others.

**FIGURE 13:**
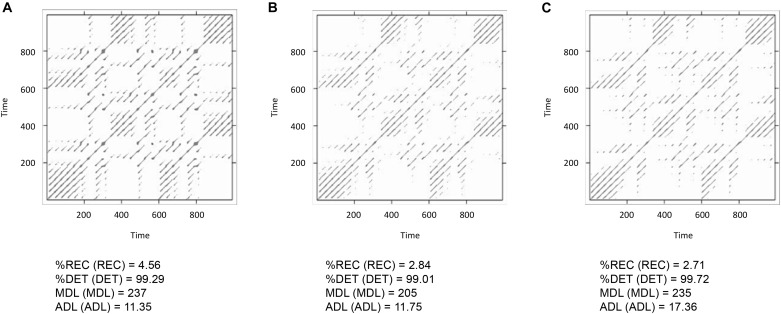
Recurrence plots and MdRQA2 results for the three dyads *lorData$x*, *lorData$y* **(A)**, *lorData$x*, *lorData$z* **(B)** and *lorData$y*, *lorData$z* **(C)** of the group.

To test this, one can run the three possible dyads as MdRQA2 (i.e., *lorData$x* with *lorData$y*, *lorData$x* with *lorData$z*, and *lorData$y* with *lorData$z*) and examine their results (see Box 6). As can be seen in Figure 13, dyad x-y seems to exhibit stronger coupling in terms of *%REC* compared to the other dyads, while dyad *y-z* seem to exhibit more stable periods of coupling in terms of *ADL* compared to the other dyads. In any case, the combination of x and z seems to exhibit the weakest coupling of the three. All, however, differ in some aspect from the “true” multivariate group-level dynamics we’ve seen when subjecting the three dimensions of the Lorenz-system simultaneous to MdRQA3 (Figure 12).

**Box 6. Running MdRQA2 on the three possible pairings of the Lorenz system.**

mdrqa2_results_xy <– mdrqa(data = as.matrix(cbind(lorData$x, lorData$y)), dims = 2, emb = 2, del = 9, norm = ‘euc’, rad = 0.2) # run MdRQA2 on the x and y dimensions of the Lorenz system

mdrqa2_results_xz <– mdrqa(data = as.matrix(cbind(lorData$x, lorData$z)), dims = 2, emb = 2, del = 9, norm = ‘euc’, rad = 0.2) # run MdRQA2 on the x and z dimensions of the Lorenz system

mdrqa2_results_yz <– mdrqa(data = as.matrix(cbind(lorData$y, lorData$z)), dims = 2, emb = 2, del = 9, norm = ‘euc’, rad = 0.2) # run MdRQA2 on the y and z dimensions of the Lorenz system

## Pitfalls and Issues

While the advantage of MdRQA lies in its applicability to groups of *n* > 2, it also has a certain number of disadvantages compared to the other techniques presented above. As of now, it is not possible to investigate leader-follower relationship with MdRQA, as for example with DCRP.

Also, there are a few issues with data preparation and parameter estimation. Currently, MdRQA parameters are estimated on the individual component time-series that enter the analysis. However, the multivariate time-series in which they are combined could exhibit different average mutual information and false-nearest-neighbor properties than the average of the individual time-series. Hence, these estimates are more uncertain. However, Wallot and Mønster (2018) have recently developed Matlab functions for the estimation of embedding parameters for multivariate time-series.

If one wants to compare the MdRQA results across different group levels (i.e., MdRQA2 with MdRQA3), one needs to keep in mind that the overall dimensionality of the time-series should be the same, or at least as similar as possible. As a rule of thumb, higher (embedding) dimensionality de-correlates the phase-space, and leads at least to fewer recurrences. Hence, if a 3-dimensional time-series is also embedded once (i.e., MdRQA3 with *emb* = 2), this results in a 6-dimensional phase-space. To compare the resulting MdRQA values with results from 2-dimensional time-series, one might have to embed these twice (i.e., MdRQA2 with *emb* = 3), which also results in a 6-dimensional phase-space (see Wallot et al., 2016a,b). However, sometimes the true dimensionality cannot be re-produced, and then trying to minimize the gap is the best that one can do.

Finally, the answer to the question of whether or not to normalize data before subjecting them to MdRQA is not straightforward. For example for the Lorenz-system, normalizing the time-series would not improve the results of the MdRQA, because the non-normalized time-series are properly scaled with regard to each other already. Similarly, when having time-series that are measured on the same scale, their absolute values might now hold meaningful information for the multidimensional dynamics – but that is not necessarily so, and normalizing the data beforehand is definitely suggested if one wants to have equal weighting of the dynamics of each individual time-series in the resulting recurrence plot and MdRQA analysis.

## A Note on Comparing Samples

The logic of using recurrence-based measures (e.g., *%REC*, *%DET*, *ADL*, *MDL, etc.*) with inferential statistics for purposes of sample comparison is not different from comparing means (or other quantities) between samples of participants – i.e., they are computed for each participant, condition, or trial, and are subsequently put into statistical models that evaluate them across these categories. However, the estimation and setting of parameters, as well as a few other issues might warrant some guiding remarks in this context. As it has been summarized in Wallot (2017) and in Wallot and Grabowski (in press) the main issues are the following:

Of course, the recurrence-based analyses above are time-series analysis techniques, so one needs to have multiple data points in order to apply the analyses. The more data points, the more reliable the measures will be, but this depends also on the dynamics in question. Additionally, because these are multivariate analyses, one needs to have at least two or more matched time-series with the same number of data points (for a tutorial introduction of applying RQA to individual time-series in R, see Wallot, 2017). With some kinds of data however – nominal data or data with strong, distinctive dynamics – as few as 10–30 data points might already be sufficient, while for others, a few hundreds or several thousands of data points are certainly desirable. Again, the most important issue is to capture the phenomenon one wants to investigate sufficiently, and to do so at an (average) sampling rate that sufficiently covers the changes in the observable over time.

When comparing data sets that compose a samples, this is usually done by first selecting a single set of parameters for all of them, then to run the respective recurrence-based analysis on each dyad (or group) and obtain the results. Hence, one would start to chart the parameters as in Table 2, and then use the same (maybe the average or maximum) value for delay and embedding dimension across all groupings in order to properly compare the resulting recurrence measures (Wallot et al., 2012). If the sample is relatively diverse in terms of the estimated parameters, one should select parameter values for delay and embedding dimension that are somewhat above the average, because recurrence-based analyses are robust against (moderate degrees) of over-embedding (Webber and Zbilut, 2005).

Similarly, a single radius parameter *r* should be picked for the whole sample: One can start again with an arbitrary value for *r*, run all pairings of data sets with this radius, and then inspect the distribution of percent recurrence (%*REC*) in the samples. As mentioned earlier, %*REC* should be low, but not too low in order to obtain meaningful results. For inter-event-times the lowest data set in the sample should have not much less than one percent of recurrence (i.e., %*REC* ≈ 1%), with the majority of the data sets being between %*REC* = 5 and 10%. For relatively deterministic time-series this figure can be lower (*%REC* between 1 and 5%, and sometimes even below that), but for very noisy data, it can be substantially higher. In any case, the radius parameter *r* might have to be adjusted a couple of times before a satisfying solution is found. If one uses categorical data, the radius should be set to 0 or to a tiny value, so that (cross-)recurrences are only based on the repetition of identical instances.

If the data sets cannot be reasonably fitted with a single radius parameter – that is, for some value of *r*, some of the data sets are at or close to %*REC* = 100%, and at the same time some are at or close to %*REC* = 0%, one can adjust the radius for each data set individually in order to keep the percentage of recurrence constant across all data sets (e.g., fixed percent recurrence of %*REC* = 5% for each data set). Of course, in this case, %*REC* needs to be omitted from the inferential statistical analysis, as it should be very similar (or the same) across pairings in all samples, but the other measures, such as %*DET*, *ADL*, and *MDL* can be still analyzed (as a stand-in for %REC, one can instead include analysis of the radius parameter *r* that is now different across pairs of groups of time-series. However, this might not yield the same results, as *r* and *%REC* do not scale linearly with each other).

If one notices great heterogeneity of the values of one of the parameters or %*REC* across the samples, one can also explore the parameter space. This means that the parameters *d*, *m*, and *r* are systematically varied to check whether the resulting solutions are stable across these variations. A common first exploration of the parameter space would be to run the analysis in question for the lowest, highest, and average values of each parameter and pick 3 different values *r* that yield low (*%REC* ≈ 1 to 3%), moderate (*%REC* ≈ 5 to 10%), and high (*%REC* ≈ 15 to 20%) percentages of (cross-)recurrence. If the resulting recurrence measures (or at least the direction of the effects observed in those measures) converge across the different parameter settings, then one can accept the occurrence of a few individual cases where *%REC* = 0 or 100%.

Another issue that arises when using recurrence-based analysis is the number of results variables produced by the analyses (e.g., *%REC*, *%DET*, *ADL*, *MDL)*. In principle, all of these variables can be dissociated, but empirically this is not always the case. As a guideline for interpretation, while relatively high *%REC* means that many individual instances in a pair or group of time-series are recurrent, *%DET* means that several of these individual instances are connected. If *%DET* is high, and *ADL* and *MDL* are low, this means we have a high, but rather homogenous cross-correlation in the time-series. If *%DET* is low and *ADL* and *MDL* are high, this means that the coupling of the time-series is very heterogeneous, and is probably composed of changing epochs of high and low correlation between the time-series. If *ADL* is high, and *MDL* is comparatively low, this could be indicative of bursting behavior, where the observed performance is composed of bursts of coupling and de-coupling.

However, the described relations between *%REC*, *%DET*, *ADL*, and *MDL* should only be seen as a rule-of-thumb, as an initial suggestion. An inspection of the associated (cross-)recurrence plots will help to interpret the shared dynamics between the time-series. As mentioned above, for inter-event-type data, many of the results variables are highly correlated, and do not actually dissociate different dynamics. In such cases, one can pick one (or a few) of the variables that seem to capture the composition of the corresponding (cross-)recurrence plot best. Alternatively, the different measures can also be subjected to principle component analysis, and the extracted component(s) can be treated as a dependent (or predictor) variable which captures generalized stability or correlation in the data – depending on the respective component loadings.

Finally, there is a yet unanswered question regarding the number of degrees of freedom in inferential statistical models when analyzing pairs of data sets. On one hand, when one is investigating all possible pairs in a sample using, for example, CRQA, one has many more observations than individual participants. Hence, such kind of analysis seems to inflate the degrees of freedom available, and adjustments for this inflation are needed. On the other and, correlations are not transitive, so it can be argued that all possible pairings actually provide a reasonably independent degree of information (except from their limits, only perfect correlations and perfect independence between data sets exist). However, this question for the current research practice has not yet received a conclusive answer.

## Conclusion and Further Readings

Overall, the combination of CRQA, DCRP, and MdRQA allows for a very detailed investigation of multivariate time-series. They allow to quantify the strength of coupling between two or more time-series, to quantify leader-follower relations between them, and to assess dynamics at different levels of composition of the observables. Recurrence-based analyses are especially suitable for time-series data with non-stationary properties; moreover, they are robust against extreme outliers and do not make any assumptions about particular distributions or particular relationships between the time-series of interest. This makes them also useful for applications in naturalistic settings, or investigations over longer time intervals. Again, we want to point out one difference between CRQA (and DCRP, accordingly) on the one hand, and MdRQA on the other hand: While the former can rely on an established procedure for proper phase-space reconstruction (if one wants to perform analysis on embedded data), it is as of now unclear how these procedures perform multivariate cases where MdRQA is used, if one does not know the embedding properties and the composition of the time-series beforehand (for a discussion, see the section “Multidimensional Recurrence Quantification Analysis”).

The current tutorial provided hands-on examples of how to run the different analysis in R, and how to utilize and interpret the results. Moreover, we outlined the current best practice for how to apply the analyses to empirical data, and in the context of sample comparisons. In addition to our recommendations, Marwan (2011) summarized general problems and pitfalls applying recurrence-based analysis that might be valuable for interested readers. Also, we can recommend the webbook edited by Riley and Van Orden (2004) for a conceptual introduction to RQA and CRQA on the webpage of the NSF, and the webpage www.recurrence-plot.tk, hosted by Norbert Marwan from the PIK Potsdam, which provides access to various other RQA-software packages, and hosts a near exhaustive bibliography of applications of recurrence-based analysis across several scientific disciplines.

## Author Contributions

SW and GL wrote the manuscript. SW coded the MdRQA-function.

## Conflict of Interest Statement

The authors declare that the research was conducted in the absence of any commercial or financial relationships that could be construed as a potential conflict of interest.
