# Supplementary material for: Analyzing Multivariate Dynamics Using Cross-Recurrence Quantification Analysis (CRQA), Diagonal-Cross-Recurrence Profiles (DCRP), and Multidimensional Recurrence Quantification Analysis (MdRQA) – A Tutorial in R
Source: Front Psychol. 2018 Dec 4;9:2232. doi: 10.3389/fpsyg.2018.02232 (PMC6288366; doi:10.3389/fpsyg.2018.02232)
Supplement: Supplementary file 1 [file Data_Sheet_1.docx]

MdRQA function translated into R from Wallot, Roepstorff, Mønster (2016). The function can also be downloaded at GitHub: https://github.com/Wallot/MdRQA

mdrqa <- function(data,emb,del,norm,rad) {

# This funciton computes a Performs Multidimensional Recurrence Quantification Analysis (MdRQA)

#

# Inputs:

#

# data = a N x M matrix, where N is the number of data points in each time series (rows) and M is the number of time-series that are analyzed together (columns)

# emb = embedding dimension parameter, where emb = 1 means no embedding, emb = 2 means creation of one surrogate copy from the original data etc.

# del = delay parameter, where del is the number of data points to be skipped for each additional embedding

# norm = norm parameter, where phase-space can be normalized by Euclidean distance 'euc', Maximum distance 'max', Minimum distance 'min', nor not 'non'

# rad = radius parameter, were rad is the distance that defines the neighborhood around a coordinate in phase-space

#

# Outputs:

#

# SizeRP =

# REC = percent recurrence

# DET = percent determinism

# ADL = average diagonal line length

# MDL = maximum diagonal line length

# DENTR = diagonal line entropy

# LAM = percent laminarity

# AVL = average vertical line length

# MVL = maximum vertical line length

# VENTR = vertical line entropy

# DIM = dimensions of input data (i.e., number of input time-series)

# EMB = embedding dimension

# DEL = delay

# NORM = phase-space normalization

# RAD = radius

# RP = recurrence plot as sparse matrix

#

# References:

#

# Wallot, S., Roepstorff, A., & Mønster, D. (2016). Multidimensional

# Recurrence Quantification Analysis (MdRQA) for the analysis of

# multidimensional time-series in MatLab and its application to group-

# level data in joint action. Frontiers in Psychology, 7, 1835.

#

# Wallot, S. & Leonardi, G. (2018). Analyzing multivariate dynamics

# using Cross-Recurrence Quantification Analysis (CRQA), Diagonal-

# Cross-Recurrence Profiles (DCRP), and Multidimensional Recurrence

# Quantification Analysis (MdRQA) – a tutorial in R. ???

#

# Version:

#

# v1.0, 13. June 2018

# by Sebastian Wallot, Max Planck Insitute for Empirical Aesthetics, Frankfurt, Germany

# Software info:

#

# The author gives no warranty for the correct functioning of the software

# and cannot be held legally accountable.

#

# This code is free and can be distributed and/or modified under the GNU

# General Public License version 2 or later (Free Software Foundation)

# Load ConnCompLabel from SDMTools and entropy from entropy

library(entropy)

library(SDMTools)

# check input variables

if (exists("data")) {

} else {

print("No data has been specified")

}

if (exists("emb")) {

} else {

emb <- 1

}

if (exists("del")) {

} else {

del <- 1

}

if (exists("norm")) {

} else {

norm <- "non"

}

if (exists("rad")) {

} else {

rad <- 1

}

# compute and store parameter settings

dims <- dim(data)[2]

PARAMETERS <- c(dims, emb, del, rad, norm)

# cut data to dims and check whether embedding needs to be performed

data <- data[,1:dims]

if (emb > 1) {

newLength <- dim(data)[1] - (emb-1)*del

tempdata <- data[1:newLength,]

for (i in seq(2,emb)) {

tempdata <- cbind(tempdata,data[(1+(del*(i-1))):(newLength+del*(i-1)),])

}

data <- tempdata

rm(tempdata)

}

# create distance matrix

RP <- as.matrix(abs(dist(data, diag = T, upper = T))*-1)

# apply norm, radius and threshold matrix

if (norm == "euc") {

RP <- RP/abs(sum(RP)/(dim(RP)[1]^2-dim(RP)[1]))

} else if (norm == "min") {

RP <- RP/abs(min(RP))

} else if (norm == "max") {

RP <- RP/abs(max(RP))

} else {

}

RP <- RP + rad

RP <- ifelse(RP < 0, 0, 1)

# calculate diagonal and vertical line distributions

diag(RP) <- 0

diagLine <- split(RP,row(RP) - col(RP))

diagHist <- 0

for (i in seq(1,(dim(RP)[1]-1)*2)) {

diagHist <- append(diagHist,tabulate(ConnCompLabel(as.numeric(unlist(diagLine[i])))))

}

diagHist <- diagHist[!diagHist %in% 0]

diag(RP) <- 1

vertLine <- split(RP,col(RP))

vertHist <- 0

for (i in seq(1,(dim(RP)[1]-1)*2)) {

vertHist <- append(vertHist,tabulate(ConnCompLabel(as.numeric(unlist(vertLine[i])))))

}

vertHist <- vertHist[!vertHist %in% 0]

# calculate MdRQA outplut

diag(RP) <- 0

RESULTS <- dim(RP)[1] # size of RP

RESULTS[2] <- 100*sum(RP)/(dim(RP)[1]^2 - dim(RP)[1]) # percent recurrence

RESULTS[3] <- 100*sum(diagHist[!diagHist %in% 1])/sum(diagHist) # percent determinism

RESULTS[4] <- mean(diagHist[!diagHist %in% 1]) # average diagonal line

RESULTS[5] <- max(diagHist) # maximum diagonal line

RESULTS[6] <- entropy(diagHist[!diagHist %in% 1]) # diagonal line entropy

RESULTS[7] <- 100*sum(vertHist[!vertHist %in% 1])/sum(vertHist) # percent laminarity

RESULTS[8] <- mean(vertHist[!vertHist %in% 1]) # average vertical line

RESULTS[9] <- max(vertHist) # maximum vertical line

RESULTS[10] <- entropy(vertHist[!vertHist %in% 1]) # vertical line entropy

diag(RP) <- 1

RP = Matrix(RP, sparse = TRUE)

output <- list(SizeRP = RESULTS[1], REC = RESULTS[2], DET = RESULTS[3], ADL = RESULTS[4], MDL = RESULTS[5], DENTR = RESULTS[6], LAM = RESULTS[7], AVL = RESULTS[8], MVL = RESULTS[9], VENTR = RESULTS[10], DIM = PARAMETERS[1], EMB = PARAMETERS[2], DEL = PARAMETERS[3], RAD = PARAMETERS[4], NORM = PARAMETERS[5], RP = RP)

return(output)

}
